# Qualitative (and Quantitative) Values of the Lung-RADS and Computed Tomography in Diagnosing Solitary Pulmonary Nodules

**DOI:** 10.3390/diagnostics12112699

**Published:** 2022-11-04

**Authors:** Lizhen Duan, Wenli Shan, Genji Bo, Guangming Lu, Lili Guo

**Affiliations:** 1 Department of Medical Imaging, The Affiliated Huai’an No.1 People’s Hospital of Nanjing Medical University, Huai’an 223300, China; 2 Department of Medical Imaging, The Affiliated Jinling Hospital, Medical School of Nanjing University, Nanjing 210002, China

**Keywords:** solitary pulmonary nodules, Lung-RADS, CT signs, ROC curve

## Abstract

**Background**: Lung-RADS classification and CT signs can both help in the differential diagnosis of SPNs. The purpose of this study was to investigate the diagnostic value of these two methods and the combination of the two methods for solitary pulmonary nodules (SPNs). **Methods**: A total of 296 cases of SPNs were retrospectively analyzed. All the SPNs were classified according to the Lung-RADS grading version 1.1. The scores of each lesion were calculated according to their CT signs. Imaging features, such as the size and margin of the lesions, pleural traction, spiculation, lobulation, bronchial cutoff, air bronchogram, vacuoles, tumor vasculature, and cavity signs, were analyzed. The imaging results were compared with the pathology examination findings. Receiver operating characteristic (ROC) curves were applied to compare the values of the different methods in differentially diagnosing benign and malignant SPNs. **Results**: The sensitivity, specificity, and accuracy of Lung-RADS grading for diagnosing SPNs were 34.0%, 94.4%, and 47.6%, respectively. The area under the ROC curve (AUC) was 0.600 (*p* < 0.001). The sensitivity, specificity, and accuracy of the CT sign scores were 56.3%, 70.0%, and 60.5%, respectively, and the AUC was 0.657 (*p* < 0.001). The sensitivity, specificity, and accuracy of the combination of the two methods for diagnosing SPNs were 93.2%, 61.1%, and 83.5%, and the AUC was 0.777 (*p* < 0.001). Conclusion: The combination of Lung-RADS classification and CT signs significantly improved the differential diagnosis of SPNs.

## 1. Introduction

Solitary pulmonary nodules (SPNs), defined as round, opaque, and smaller than 3 cm in diameter, are one of the most common findings on chest computed tomography (CT) scans [1,2]. SPNs mainly comprise malignant lesions, such as primary and metastatic tumors, benign tumors, and infectious lesions [3]. Early stage lung cancer is often characterized by SPNs; therefore, an important primary step in the management of patients with lung nodules is estimating the probability of malignancy [4]. The National Lung Screening Trial (NLST), initiated in 2002, showed a 20% relative reduction in mortality due to lung cancer with three rounds of low-dose CT screening when compared with radiography [5,6]. In the NLST study, the false-positive rate was as high as 24% [6]. Excessive false-positive rates and over-diagnoses of lung cancer may lead to over-treatment [7]. 

To decrease the false-positive rates of CT lung screening and to optimize CT screening for SPN detection, the American College of Radiology (ACR) endorsed the Lung CT Screening Reporting and Data System version 1.1 (Lung-RADS 1.1) on 2 January 2019 [8]. Lung-RADS is divided into six categories based on CT findings; each category is associated with the risk of primary lung malignancy and specific recommendations for examination [9]. According to Lung-RADS, the degree of malignancy increases as the nodule category increases from 1 to 4X [10]. A study by Brady et al. reported that the application of the ACR Lung-RADS increased the positive predictive value of CT lung screening by a factor of 2.5% to 17.3% without increasing the false-negative results [11].

The diagnosis of SPNs primarily depends on the clinical parameters and imaging findings. The clinical parameters include age, smoking history, family history, exposure, associated lung diseases, and clinical history [12]. The imaging features of CT include size, margin, calcification, pleura traction sign, spiculation, lobulation, bronchial cutoff sign, tumor vasculature, vacuole, and cavity. These CT signs of the morphology of SPNs are important parameters in distinguishing benign from malignant SPNs [13]. In a recent study, the CT quantitative parameters, which include diameter, volume, and solid, were able to predict the invasiveness of lung pure ground-glass nodules (pGGNs) [14]. In addition, many research studies have demonstrated that lung cancer is associated with mutations in multiple genes, including p63, EGFR, ALK, CGP, BRCA1/2, and so on [15,16,17,18]. Understanding the molecular pathways and predictive potential of these genes is also critical to advancing the differential diagnosis of benign and malignant SPNs. However, the cost of gene-related biomarker tests limits the wide application of this research to clinical practice.

Patients often undergo repeated examinations due to excessive anxiety [19]. Over-diagnoses is often associated with the anxiety, additional costs, and morbidity caused by treatment. Data from the NSLT demonstrated that 18.5% of SPNs diagnosed by CT screening were over-treated [20]. Early diagnosis of SPNs is of great significance to save medical resources and provide appropriate clinical follow-up or surgical advice. In this study, the CT findings of 296 patients with SPNs were collected and analyzed to verify the diagnostic efficacy of Lung-RADS grading, CT signs, and the combination of these two methods in the clinical setting for the diagnosis of SPNs.

## 2. Materials and Methods

### 2.1. Patient Selection

We retrospectively collected the data of 296 patients (127 males; 169 females; age, 26–78 years; average age, 55.6 ± 9.7 years) with SPNs in Huai’an No.1 People’s Hospital between October 2017 and September 2019. The CT images were extracted from a picture archiving and communication system. The inclusion criteria were histologically confirmed solitary pulmonary nodules (≤3 cm), complete chest CT imaging data without motion artifacts, and no other clinical intervention. The exclusion criteria were lesion size >3 cm, history of lung cancer, and the presence of other tumors. Lung nodules are divided into benign nodules and malignant nodules. Benign nodules include adenoma, papilloma, chronic hyperplasia and hamartoma, etc. Malignant nodules include glandular precursor lesions, such as atypical adenoma hyperplasia (AAH) and adenocarcinoma in situ (AIS), and adenocarcinoma, such as minimally invasive adenocarcinoma (MIA) and invasive adenocarcinoma (IAC) [21]. Of the 296 cases, 90 (30.4%) were benign and 206 (69.6%) were malignant. Thirty-six patients had coughing, expectoration of sputum, and blood in sputum as the first clinical symptoms. Eighteen patients also had chest symptoms, such as chest pain and chest distress. Most of the cohort (247 patients; 83.4%) occasionally underwent physical examinations. This retrospective study was approved by the local Institutional Review Board and was conducted in accordance with the ethical standards of the Declaration of Helsinki.

### 2.2. CT Imaging Acquisition 

CT imaging was performed for the patients with a 64-detector row dual source CT scanner (SIEMENS SOMATOM Definition Flash; Siemens, Erlangen, Germany), using the following parameters: slice thickness, 1 mm; acquisition field of view (FOV), 500 mm; beam pitch, 1.35 or 1.375; matrix, 512 × 512; gantry speed, 0.6 s per rotation; tube voltage, 80~140 kV; and tube current, 60~100 mA. The images are displayed under the lung window (width, 1200HU (Hounsfield units); level, −600 HU) and the mediastinal window (width, 350 HU; level, 50 HU). The scanning areas ranged from the apex to the bottom of the lung using the Care Dose 4D mode. To facilitate the observation of lesion characteristics, the original data were transferred to the specific post-processing workstation (Syngo Via software) for reconstruction. Multiplanar reconstruction (MPR), volume reconstruction (VR), and maximum density projection (MIP) were performed. Sagittal and coronal images were also reconstructed.

### 2.3. Analysis of CT Imaging 

Two thoracic radiologists (with 10 years and 15 years of experience performing chest imaging, respectively) were blinded to the clinical and pathological data and examined all images using both mediastinal (width, 350 HU; level, 50 HU) and lung (width, 1200 HU; level, −600 HU) windows. They reached an agreement based on the assessment of the analyzable nodules. The radiologists had certain biases based on their individual expertise; therefore, we evaluated the reproducibility of the semantics scores of the two radiologists (Table 1). Additionally, we randomly selected 40 cases and asked a third radiologist (G.L.) to score the semantics.

The characteristics involved in this study included the margins and signs of the lesions. The majority of the radiologists habitually defined margin irregularity, pleural traction sign, spicule sign, lobulation sign, bronchial cutoff sign, tumor vasculature sign, air bronchogram sign, vacuole sign, and cavity sign as malignant signs [22,23,24,25]. Calcification and fat were considered benign signs [26,27]. Malignant signs were given a score of 1, and benign signs were given a score of −1. 

The nodules were divided into three categories based on the Lung-RADS 1.1: pGGNs (85 cases), part-solid nodules (PSNs) (61 cases), and solid nodules (SNs) (150 cases). Additionally, the lesions were graded according to their size and signs. The nodule and the solid portion were measured by averaging the longest and shortest diameters of the maximum cross-section of the lesion in the lung windows. The following nodule signs were observed: the margin of the lesion (smooth or irregular); pleural traction sign; spicule sign; lobulation sign; bronchial cutoff sign; air bronchogram sign; tumor vasculature sign; vacuole sign; cavity sign; and benign signs (i.e., fat and calcification). All the nodules were classified as grade 1 to 4X. An agreement regarding the results was reached when differences existed. 

### 2.4. Statistical Analysis

The data were analyzed by IBM SPSS Statistics 22 Developer software. A kappa test was used to verify the consistency of the semantics scores between the two radiologists. The chi-square test was used to compare the different characteristics of the benign and malignant groups. Statistical differences between the groups with regard to age, lesion size, and solid portion size in each group were analyzed by using the independent sample *t* test. The Kruskal–Wallis test was used to analyze the Lung-RADS classifications and nodule categories of the benign and malignant groups. The accuracy of the Lung-RADS grading, the CT signs used for scoring, and a combination of these two methods for diagnosing SPNs was evaluated using receiver operating characteristic (ROC) curves. The cutoff value was obtained from the highest point of the Youden index. The areas under the ROC curve (AUCs) were used to estimate the diagnostic value of each method. An AUC greater than 0.9 demonstrated excellent diagnostic efficacy. An AUC between 0.7 and 0.9 indicated good diagnostic efficacy. Poor diagnostic efficacy was indicated by an AUC between 0.5 and 0.7. A logistic regression analysis was performed to compare the differences in the AUCs (MedCalc software). *p* < 0.05 was considered statistically significant. 

## 3. Results

### 3.1. Pathological Diagnosis, CT Signs, and Scores

Ninety-five patients had a history of smoking, twenty-four patients had chronic obstructive pulmonary disease, and thirty-five patients had emphysema. Pathologically, 90 of the 296 cases were benign nodules. Among these benign cases, there were 60 cases of chronic organic inflammation, other benign lung tumors, and so on. Furthermore, 206 lesions were malignant. Among these, there were 50 cases of glandular precursor lesions, including atypical adenoma hyperplasia (AAH) and adenocarcinoma in situ (AIS), and 156 cases of adenocarcinoma, including minimally invasive adenocarcinoma (MIA), and invasive adenocarcinoma (IAC) (Table 2). 

**Figure 1:**
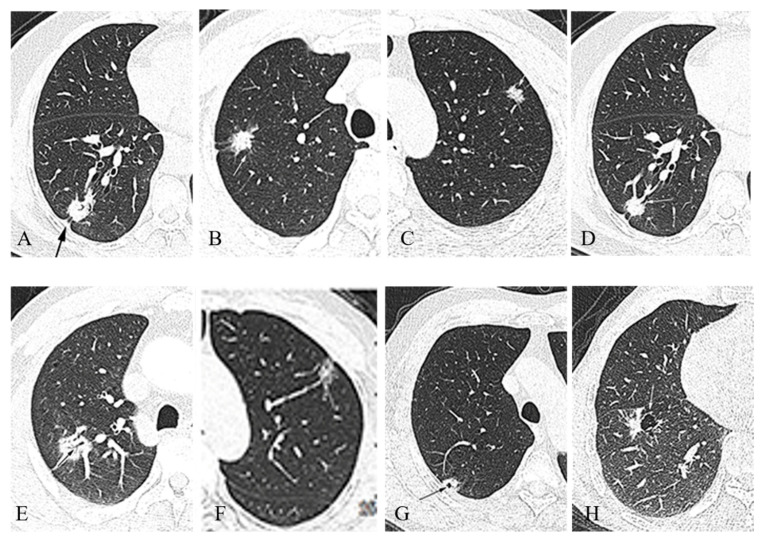
Display of common malignant signs: (**A**) pleura traction sign (black arrow); (**B**) spicule sign (white arrow); (**C**) lobulation sign; (**D**) bronchial cutoff sign; (**E**) air bronchogram sign (black arrow); (**F**) tumor vasculature sign; (**G**) vacuole Sign (black arrow); (**H**) cavity sign (white arrow).

In this study, the size of the nodules increased with the increase in the degree of malignancy (*p* < 0.001). In the PSNs, the solid portion successively increased in AAH, AIS, MIA, and IAC (*p* < 0.05) (Table 3). The results of the CT evaluations regarding the distribution of the benign and malignant pulmonary nodules and the CT scores are shown in Table 4. The malignant signs were scored as 1; the benign signs were scored as −1. After statistical analysis, incidences of irregular edges, bronchial cutoffs, vacuoles, and tumor vasculature signs in the malignant group were higher than those in the benign group (*p* < 0.05). The signs of pleural traction, spicules, lobulation, and air bronchogram signs were not statistically significant in the benign and malignant groups (*p* > 0.05). There were few cases of calcification, fat, and cavities in our study; therefore, there were no statistical values for these three signs. The CT scores of the benign and malignant pulmonary nodules are provided in Table 5. We can see that the CT score of −1 indicates purely benign nodules, the cumulative CT score of 8 points indicates purely malignant nodules, and the score of 4 points is a threshold. The cases of benign and malignant nodules within 4 points were similar. After more than 4 points, the cases of malignant nodules were more than those of benign nodules. The CT findings of the typical malignant signs are shown in Figure 1.

### 3.2. Values of Lung-RADS Grading, CT Signs, and a Combination of these Methods for Diagnosing SPNs

**Figure 2:**
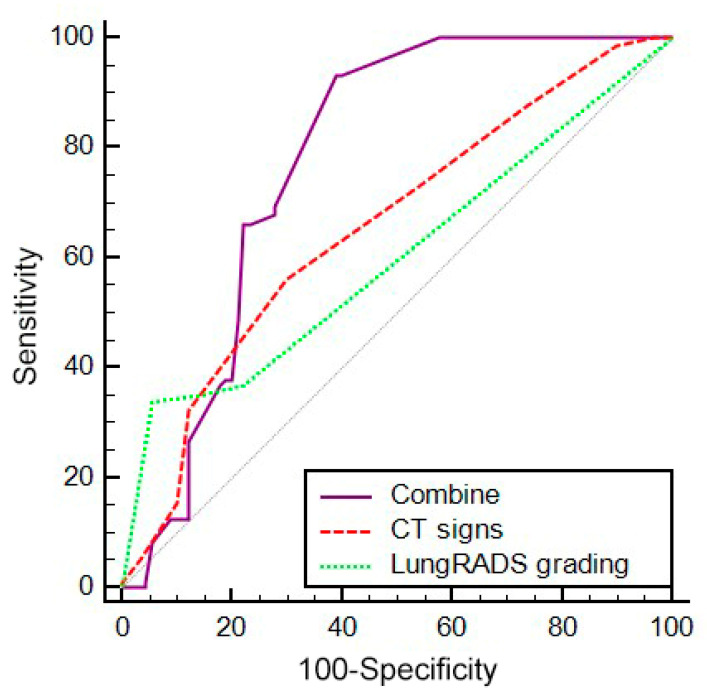
ROC curve of the diagnostic value of Lung-RADS grading, CT signs, and a combination of these two methods.

Each lesion was reclassified according to the Lung-RADS criteria (Table 6). Seventy-five cases were grade 2; ten cases were grade 3; eleven cases were grade 4A; and two hundred cases were grade 4B/X. The ROC curves were utilized to analyze the efficacy of the Lung-RADS grading, the CT signs, and a combination of both for diagnosing benign and malignant SPNs. The AUCs of the three methods were 0.600, 0.675, and 0.777, respectively. There were no significant differences in the diagnostic efficacy of the Lung-RADS grading and the CT signs (Z = 1.161; *p* = 0.2457). The diagnostic efficacy of the combined methods for SPNs was higher than that of the Lung-RADS grading (Z = 5.404; *p* < 0.001) and that of the CT signs (Z = 3.893; *p* < 0.001) (Figure 2). 

According to the Youden index, the diagnostic threshold of each scheme was determined, and the sensitivity, specificity, and accuracy were calculated. The cutoff value of the CT signs was 4 points, and the cutoff value of the Lung-RADS grading was category 3. Regarding the CT signs, the sensitivity, specificity, and accuracy were 56.3%, 70.0%, and 60.5%, respectively, and the AUC was 0.657 (*p* < 0.001). Detailed reports of each CT score are presented in Table 7. The sensitivity, specificity, and accuracy of the Lung-RADS grading for diagnosing the SPNs were 34.0%, 94.4%, and 47.6%, respectively, and the AUC was 0.600 (*p* < 0.001). The thorough results of the Lung-RADS categories 2 to 4B/X for diagnosing the SPNs are shown in Table 8. When the two methods were combined, the sensitivity, specificity, and accuracy for diagnosing the SPNs were 93.2%, 61.1%, and 83.5%, respectively, and the AUC was 0.777 (*p* < 0.001).

## 4. Discussion

The ACR formulated the Lung-RADS grading criteria to standardize the baseline screening, clinical reporting, and therapeutic recommendations for lung cancer [28]. The criteria are primarily based on the size of the lesion and the content of the solid components. The size and growth patterns of SPNs are vital factors in malignancy evaluation. Obviously, malignancy increases as the nodule diameter increases. The density of the ground-glass nodules (GGNs) was slightly higher than that of the normal lung parenchyma; meanwhile, the normal lung structures, such as the bronchus and blood vessels, existed in the nodules. SSNs are described as solid components in GGNs. When the solid components are located in the center of the lesion, the consolidation surrounded by ground-glass opacity is often called the halo sign [29]. This sign can be seen in both benign and malignant lesions. In malignant nodules, the halo sign is caused by the proliferation of tumor cells along the entire alveolar wall during local diffusion without interstitial or vascular invasion. These nodules usually persist during follow-up. However, partially solid nodules usually have a significantly higher risk of malignancy than solid nodules [30]. One longitudinal study reported that the progression of malignant solitary pulmonary nodules can be characterized only by an increase in the solid components of the lesion without an increase in the diameter or volume [31]. An ROC curve analysis performed to differentiate benign from malignant nodules indicated that the sensitivity and specificity for tumor size (cutoff, 11 mm) were 95.8% and 46.8%, respectively, and the AUCs were 0.75 and 0.77, respectively [32]. In this study, the nodule size increased with the increased degree of malignancy, and there was a high correlation between the volume of the solid components in the GGNs and the degree of malignancy. Lung-RADS grades 2 to 4A had a lower sensitivity and a higher specificity, thus indicating high false-negative rates and low false-positive rates. Hence, the risk of missed diagnosis was increased. The sensitivity, specificity, and accuracy of diagnosis in Lung-RADS 4B/X lesions were 100%, 0%, and 65.0%, respectively. The diagnostic specificity of Lung-RADS grade 4B/X was low, which may have increased the false-positive rate because some malignant signs were also present in the benign nodules. Therefore, the benign nodules (with malignant signs) may be mistaken for malignant lesions, resulting in premature intervention and over-treatment. The AUC was 0.600 (*p* < 0.05).

There were certain limitations in the differential diagnosis of the SPNs in the present study. The size and the solid components of lesions are particularly valuable in the differential diagnosis of benign and malignant pulmonary nodules, but the morphology of SPNs should not be underestimated [27,33,34]. In a study covering 112 GGNs (82 malignant and 30 benign), the vascular convergence signs, the lobulation, the spiculation, and the pleural tags were risk factors for malignancy, with a sensitivity, specificity, and accuracy of 60.0%, 93.5%, 83.3%, and 87.2%, respectively [35]. A smooth margin and a fat popcorn-like calcification indicated that the nodule was benign. More features related to malignancy were described, including margin irregularity, spicules, lobulation, pleural traction, bronchial cutoff, air bronchogram, tumor vascularity, vacuoles, and cavity sign [33,36,37,38]. Therefore, in this study, irregular borders, pleural traction, spicules, lobulation, bronchial cutoff, air bronchogram, vacuoles, tumor vascularity, and cavity signs were defined as malignant signs, whereas calcification and fat were defined as benign signs. After statistical analysis, the incidences of irregular borders, bronchial cutoffs, vacuoles, and tumor vasculature signs in the malignant group were higher than those in the benign group (*p* < 0.05), which was partly consistent with the aforementioned views. Signs of pleural traction, spicules, lobulation, and air bronchogram were also visible in the inflammatory lesions [39]. In our study, chronic inflammation accounted for 66.7% cases in the benign group, possibly explaining why the pleural traction, spicules, lobulation, and air bronchogram signs were not statistically significant in the benign and malignant groups. There were few cases of calcification, fat, and cavities in our study; therefore, there were no statistical values for these three signs. 

In this study, when the CT score was more than 3 points, the sensitivity, specificity, and accuracy of the CT signs for diagnosing the SPNs were 75.9%, 38.5%, and 59.5%, respectively (*p* < 0.001). The diagnostic efficacy of the CT signs was higher when there were more than three malignant signs in the SPNs. When the CT score was 8 points, the sensitivity, specificity, and accuracy of the CT signs for diagnosing the SPNs were 97.0%, 100%, and 100%, respectively (*p* < 0.001). When there were more CT signs in the SPNs, the lesions were more likely to be malignant and the specificity and accuracy were improved. There were no significant differences in the diagnostic efficacy of the CT signs and the Lung-RADS grading (*p* > 0.05). The sensitivity, specificity, and accuracy of the combined methods were 93.2%, 61.1%, and 83.5%, respectively, and the AUC was 0.777 (*p* < 0.001). The value of the combined methods was greater than that of the Lung-RADS grading (Z = 5.404; *p* < 0.001) and that of the CT signs (Z = 3.893; *p* < 0.001). CT signs and Lung-RADS grading have limitations in the differential diagnosis of SPNs. As we all know, there are some malignant signs in benign nodules, and benign nodules (with malignant signs) may be mistaken for malignant lesions. In clinical studies, the differential diagnosis of benign and malignant SNPs is still a challenging task limited by the experience and subjectivity of the radiologists. Furthermore, according to the Lung-RADS grading, nodules of grade 3 or 4, once they have other characteristics or imaging findings which increase the suspicion of malignant tumors, would be graded as 4X [10]. Therefore, many inflammatory nodules with malignant signs are evaluated as 4X and undergo puncture or surgery. When the two methods were combined, the size, solid components, and morphological features of the lesions were comprehensively analyzed, thus providing more meaningful and objective imaging data that could be used to determine the diagnosis. Therefore, combining the two methods can significantly improve the diagnostic efficacy. Whether CT signs can confirm the features of the Lung-RADS 4X nodules and result in improved data still needs to be verified by further research.

The Lung-RADS classification is mainly used for groups at high risk for lung cancer or with risk factors such as emphysema, chronic obstructive pulmonary disease, and occupational and environmental factors. The major limitation of the study was the clinical application of the methods. The bias involved in the Lung-RADS grading of benign and malignant SPNs is inevitable. Our study revealed the accuracy of CT signs when diagnosing SPNs, and it revealed that their use is similar to that of the Lung-RADS classification. However, the combination of the two methods resulted in better diagnostic performance and diagnostic efficacy and can help clinicians to choose reasonable treatment measures. There may have been several limitations to our study: firstly, as all the cases were pathological proven nodules, the proportion of malignant cases was inevitably higher in the selected cases. Secondly, the small sample of cases included may not have been enough to contain all the benign and malignant features. 

In conclusion, the Lung-RADS classification and CT signs were significant for the differential diagnosis of benign and malignant pulmonary nodules. The diagnostic efficacy of the CT signs was similar to that of the Lung-RADS classification. The combination of these two methods can significantly improve the differential diagnosis of SPNs.

## Figures and Tables

**Table 1:** Agreement for semantic features determined by two readers.

CT Features	Kappa
Contour	0.820
Pleural traction sign	0.951
Spicule sign	0.949
Lobulation sign	0.880
Bronchial cutoff sign	0.857
Air bronchogram sign	0.952
Tumor vasculature sign	0.872
Vacuole sign	0.784
Cavity sign	0.664
Fat	1.000
Calcification	1.000

**Table 2:** Subject characteristics.

Characteristics	Benign Cases (n = 90)	Malignant Cases (n = 206)	All (n = 296)	*p*
Age (y) *	56.6 ± 9.4	53.4 ± 9.9	55.6 ± 9.7	0.005 ^†^
Men, n (%)	56 (62.2)	71 (34.5)	127 (42.9)	<0.001
Smoking, n (%)	37 (41.1)	58 (28.2)	95 (32.1)	0.035
History of COPD, n (%)	6 (6.7)	18 (8.7)	24 (8.1)	0.550
Emphysema, n (%)	9 (1.0)	26 (12.6)	35(11.8)	0.522
Lung-RADS classification, n (%)				0.001 ^‡^
2	5 (5.6)	70 (34.0)	75 (25.3)	
3	8 (8.9)	2 (1.0)	10 (3.4)	
4A	7 (7.8)	4 (1.9)	11 (3.7)	
4B/X	70 (77.8)	130 (63.1)	200 (67.6)	
Category, n (%)				<0.001
Chronic organic inflammation	60 (66.7)	-	60 (20.3)	
Hamartoma	12 (13.3)	-	12 (4.1)	
Tuberculosis	6 (6.7)	-	6 (2.0)	
Fungal infection	4 (4.4)	-	4 (1.4)	
Lymphoid hyperplasia	3 (3.3)	-	3 (1.0)	
Sclerosing hemangioma	4 (4.4)	-	4 (1.3)	
Fibromatosis	1 (1.1)	-	1 (0.3)	
AAH	-	24 (11.7)	24 (8.1)	
AIS	-	26 (12.6)	26 (8.8)	
MIA	-	22 (10.7)	22 (7.4)	
IAC		134 (65.0)	134 (45.3)	
Nodule size (mm) *	12.7 ± 5.8	14.0 ± 5.8	13.6 ± 5.8	0.018 ^†^
Nodule category, n (%)				<0.001 ^‡^
pGGN	11 (12.2)	74 (35.9)	85 (28.7)	<0.001
mGGN	6 (6.7)	55 (26.7)	61 (20.9)	<0.001
SSN	73 (81.1)	77 (37.4)	150 (50.7)	<0.001

COPD = chronic obstructive pulmonary disease. * Data are means ± standard deviation. ^†^ Independent sample *t* test. ^‡^ Kruskal–Wallis test. Except when indicated, the method of data analysis was the Pearson x^2^ test.

**Table 3:** Size of nodules and solid portion in each malignant group.

Characteristic	AAH (n = 24)	AIS (n = 26)	MIA (n = 22)	IAC (n = 134)	*p* ^‡^
Size of nodules	9.8 ± 3.9	9.9 ± 3.4	13.9 ± 6.9	15.6 ± 5.4	<0.001
Size of solid portion in mGGN	6.5 ± 2.6	6.9 ± 2.3	9.0 ± 5.5	11.3 ± 3.4	<0.05

^‡^ Kruskal–Wallis test.

**Table 4:** Distribution of CT signs and CT score of each sign.

Signs	Calcification	Fat	Margin Irregularity	Pleural Traction Sign	Spicule Sign	Lobulation Sign	Bronchial Cutoff Sign	Air Bronchogram Sign	Tumor Vasculature Sign	Vacuole Sign	Cavity Sign
CT score	−1	−1	1	1	1	1	1	1	1	1	1
Benign	2	6	70	50	24	22	4	36	38	6	1
Malignant	0	0	194	125	56	55	38	92	166	35	1
*p*			<0.001	>0.05	>0.05	>0.05	<0.05	>0.05	<0.001	<0.05	

**Table 5:** Cumulative CT scores for benign and malignant solitary pulmonary nodules.

CT Score	−1	0	1	2	3	4	5	6	7	8
Benign	3	6	15	17	22	16	2	6	3	0
Malignant	0	3	23	29	35	49	35	21	9	2

**Table 6:** Lung-RADS classification.

Classification	Lung-RADS Category	Criteria
Benign	2	Perifissural nodule(s) <10 mm Solid nodule(s): <6 mm; new: <4 mm
		Part-solid nodule(s): <6 mm total diameter on baseline screening
		Non-solid nodule(s) (GGN): <30 mm or ≥30 mm and unchanged or slowly growing
		Category 3 or 4 nodules unchanged for ≥3 months
Probably benign	3	Solid nodule(s): ≥6 to <8 mm at baseline or new 4 mm to <6 mm
		Part-solid nodule(s): ≥6 mm total diameter with solid component <6 mm or new <6 mm total diameter
		Non-solid nodule(s) (GGN): ≥30 mm on baseline CT or new
Suspicious	4A	Solid nodule(s): ≥8 to <15 mm at baseline or growing <8 mm or new 6 to <8 mm
		Part-solid nodule(s): ≥6 mm with solid component ≥6 mm to <8 mm or with a new or growing <4 mm solid component
		Endobronchial nodule
Malignant	4B/X	Solid nodule(s): ≥15 mm or new or growing, and ≥8 mm
		Part-solid nodule(s) with a solid component ≥8 mm or a new or growing ≥4 mm solid component
		Category 3 or 4 nodules with additional features or imaging findings that increase the suspicion of malignancy

Lung-RADS™ Version 1.1 assessment categories. Release date: 2 January 2019.

**Table 7:** Sensitivity, specificity, and accuracy of CT scores for diagnosing SPNs.

CT Score	−1	0	1	2	3	4	5	6	7	8
Sensitivity	1.00	1.00	0.99	0.87	0.73	0.56	0.33	0.16	0.05	0.97
Specificity	0.00	0.03	0.10	0.27	0.46	0.70	0.88	0.90	0.97	1.00
Accuracy	1.00	0.67	0.39	0.37	0.39	0.75	0.95	0.78	0.75	1.00

**Table 8:** Sensitivity, specificity, and accuracy of Lung-RADS 1-4B/X for diagnosing SPNs.

Lung-RADS Grading	2	3	4A	4B/X
Sensitivity	0.34	0.35	0.37	1.00
Specificity	0.94	0.86	0.78	0.00
Accuracy	0.07	0.20	0.36	0.65

## Data Availability

The data used to support the findings of this study are available from the corresponding author upon request.
